# Elevated levels of circulating plasma sPDGFRβ in cognitively impaired *APOE4* carriers

**DOI:** 10.1007/s11357-026-02291-y

**Published:** 2026-05-12

**Authors:** Abhay P. Sagare, Trevor Lohman, Ivan Ramirez, Elizabeth B. Joe, Michael G. Harrington, Helena C. Chui, Lon S. Schneider, Hussein N. Yassine, John M. Ringman, Arthur W. Toga, Daniel A. Nation, Berislav V. Zlokovic

**Affiliations:** 1 Department of Physiology and Neuroscience, Zilkha Neurogenetic Institute, Keck School of Medicine, University of Southern California, 1501 San Pablo Street, Los Angeles, CA 90089 USA; 2 Leonard Davis School of Gerontology, Keck School of Medicine, University of Southern California, 3715 McClintock Avenue, Los Angeles, CA 90089 USA; 3 Alzheimer’s Disease Research Center, Keck School of Medicine, University of Southern California, Los Angeles, CA USA; 4 Department of Neurology, Keck School of Medicine, University of Southern California, Los Angeles, CA USA; 5 Department of Psychiatry and Behavioral Sciences, University of Southern California, Los Angeles, CA USA; 6 Laboratory of Neuro Imaging (LONI), USC Stevens Neuroimaging and Informatics Institute, Keck School of Medicine, University of Southern California, Los Angeles, CA USA

**Keywords:** sPDGFRbeta, Pericyte injury, Blood-brain barrier, APOE4, Alzheimer's disease

## Abstract

Increased levels of cerebrospinal fluid (CSF) soluble platelet-derived growth factor receptor-β (sPDGFRβ), a marker of blood–brain barrier (BBB)-associated pericyte cell injury, have been shown to correlate with increased BBB permeability and severity of Alzheimer’s disease (AD) pathology. It has been also shown that increased CSF sPDGFRβ levels can predict cognitive dysfunction in *APOE4* carriers bearing the main susceptibility gene for AD. Whether plasma sPDGFRβ levels are also elevated in *APOE4* carriers has not been explored. Here, we analyzed plasma samples for sPDGFRβ levels, Ab_42/40_ ratio, and pTau217 in 115 *APOE3* homozygotes and *APOE4* carriers with Clinical Dementia Rating (CDR) score of 0 and 0.5. Our data show that plasma sPDGFRβ levels are increased in *APOE4* carriers with cognitive impairment compared to *APOE4* carriers that are cognitively unimpaired as well as relative to cognitively impaired *APOE3* carriers even after controlling for plasma Aβ_42/40_ ratio and pTau217. Thus, our data suggest that plasma sPDGFRβ is a useful biomarker of brain pericytes/BBB damage in *APOE4* carriers.

## Introduction

The blood–brain barrier (BBB) is composed of the tight-junction sealed endothelium of the central nervous system microvasculature which limits entry into the brain of blood-derived neurotoxic molecules, environmental toxins, microorganisms such as viruses, bacteria and fungi, and circulating cells [1, 2]. Under physiological conditions, the BBB provides brain cells with nutrients, key substrates for DNA and RNA synthesis, and regulatory molecules from blood via substrate-specific transport mechanisms. At the same time, the BBB provides a key clearance function for the brain by eliminating metabolic waste products and neurotoxic molecules produced by brain metabolism to blood [1, 2]. The BBB-associated pericyte cells critically maintain BBB integrity, and their loss is associated with BBB breakdown and dysfunction [3–6].

BBB breakdown and/or dysfunction associated with deposition into the brain of blood-derived neurotoxic molecules, such as fibrinogen and iron-containing protein deposits (e.g. hemosiderin) [7], loss of endothelial cells, pericytes and activation of BBB surrounding astrocytes and pericapillary microglia cells, have been described by pathological studies in several neurodegenerative disorders including Alzheimer’s disease (AD) [1, 8, 9]. It has been suggested that pathological changes triggered by BBB breakdown and neurovascular dysfunction can initiate and/or contribute to neuronal and synaptic dysfunction, inflammatory response, neurodegeneration, and cognitive impairment and may play a role in the pathogenesis of AD and related disorders [10–13].

Recent neuroimaging studies have suggested that BBB leaks occur during normal aging and are related to age-dependent cognitive decline, particularly in individuals developing loss of memory retrieval and mild cognitive impairment (MCI) [14–19]. It has been also shown that changes in BBB in individuals with MCI and AD correlated with cognitive decline independently of amyloid-β (Aβ) and tau AD biomarkers measured either in the cerebrospinal fluid (CSF) or brain [19–21]. Moreover, individuals bearing apolipoprotein E (*APOE4*), the major susceptibility gene for AD [22] have been shown to develop accelerated BBB breakdown and loss of pericytes [23–27] compared to individuals that are homozygous for *APOE3* isoform (ε3/ε3) that carries lower risk for AD [22]. Furthermore, recent neuroimaging studies have demonstrated that *APOE4* carriers (ε3/ε4 and ε4/ε4) compared to *APOE3* homozygotes (e3/e3) develop accelerated BBB dysfunction which is further enhanced with cognitive impairment [19, 20, 28, 29].

In agreement with neuroimaging studies, it has been shown that increased CSF levels of soluble PDGFRβ (sPDGFRβ), a marker of BBB-associated pericyte cell injury, correlate with BBB permeability changes in individuals with MCI [14, 20, 21] as well as in AD patients and in individuals with increased AD biomarkers [30–37]. It has been also demonstrated that increased CSF sPDGFRβ levels can precede and/or predict later cognitive decline in *APOE4* carriers [20, 38]. Another study reported that CSF sPDGFRβ levels were positively correlated with serum sPDGFRβ levels in neurologically normal matched samples [30]. However, whether circulating sPDGFRβ can be used as a biomarker of pericyte/BBB damage in individuals at high risk for AD such as *APOE4* carriers has not been explored.

Therefore, the present study examined plasma sPDGFRβ levels in *APOE3* homozygotes and *APOE4* carriers in relation to cognitive impairment in individuals that developed cognitive impairment with Clinical Dementia Rating (CDR) score of 0.5 compared to those that were clinically unimpaired with CDR score of 0. Additionally, we studied whether plasma Aβ_42/40_ ratio and plasma pTau217 can influence the predictive value of plasma sPDGFRβ biomarker. Our data suggest that plasma sPDGFRβ is a useful biomarker of brain pericytes/BBB damage even after controlling for plasma Aβ_42/40_ ratio and pTau217 biomarkers.

## Materials and methods

### Participants

Participants were recruited from the community and the University of Southern California (USC) Alzheimer’s Disease Research Center (ADRC) if they were living independently and between the ages of 55 and 89. Study was approved by USC Institutional Review Board, and participants gave written informed consent for blood draw. Study exclusions included a history of clinical stroke, dementia, moderate-to-severe traumatic brain injury, major psychiatric or neurologic illness, organ failure or major systemic illness, or active medications or other medical conditions that could impact cognitive function. Participants were not excluded based on the presence of stable, controlled cardiovascular risk factors, such as hypertension, hyperlipidemia, coronary artery disease, or type 2 diabetes. Demographic characteristics of 115 participants in sPDGFRβ study is shown in Table 1. Participants were grouped according to *APOE* genotype and the Clinical Dementia Rating (CDR) global score (0–0.5), and age, sex, level of education, vascular risk factors (VRF), plasma Aβ_42/40_ ratio, and plasma pTau217 were studied as variables.

**Table 1:** Demographic characteristics of the participants in sPDGFRβ study grouped according to *APOE* genotype and the Clinical Dementia Rating (CDR) score

APOE Genotype		*APOE3*	*APOE4*	*APOE3*	*APOE4*
CDR Score		0	0	0.5	0.5
Plasma sPDGFRβ	n	50	34	15	16
Age	Mean (SD)	69.75 (6.4)	67.06 (5.9)	70.93 (8.4)	71.56 (6.5)
Female	n (%)	19 (38.0)	15 (44.1)	9 (60.0)	5 (31.3)
Education	Mean (SD)	16.66 (2.4)	16.5 (2.3)	15.27 (3.4)	16.31 (2.5)
VRF	n 0–1/2 +/NA	30/20/0	17/17/0	7/8/0	7/8/1
Plasma Aβ_42/40_ ratio	Mean (SD)	.093 (.009)	.090 (.007)	.092 (.013)	.084 (.007)
Plasma pTau217	Mean (SD)	4.61 (4.05)	5.47 (5.25)	7.25 (5.48)	8.93 (5.56)

The variables include age, sex, level of education, vascular risk factors (VRF), plasma Aβ_42/40_ ratio and plasma pTau217. *APOE3* denotes *APOE3* homozygotes (e3/e3); *APOE4* denotes *APOE4* carriers (e3/e4 and e4/e4). Note: SD = standard deviation

### Vascular risk factors

Each participant underwent a physical examination, blood tests, and interviews to determine presence of vascular risk factors (VRFs). The presence of VRFs were defined based on classification from the Framingham Stroke Risk Profile [39] and included a history of cardiovascular disease (heart failure, angina, stent placement, coronary artery bypass graft, intermittent claudication), hypertension, hyperlipidemia, type 2 diabetes, atrial fibrillation, and transient ischemic attack or minor stroke. The total burden was defined by the sum of these specific risk factors. Based on prior studies linking 2 + VRFs vs. 0–1 VRFs to cerebrovascular pathology, participants were grouped by total VRF burden of 0–1 vs. 2 + consistent with prior studies [40, 41].

### Venipuncture and separation of plasma

The participant's blood collected after overnight fasting in EDTA-coated tubes was centrifuged at 2,000 g and 4 °C for 10 min, aliquoted in polypropylene tubes, and stored at −80 °C until analyses. The Buffy coat was collected in a separate tube for DNA isolation and *APOE* genotyping.

### *APOE* genotyping

Briefly, DNA was extracted from buffy coat followed by *APOE* genotyping by polymerase chain reaction (PCR)-restriction fragment length approach [42]. Participants were stratified based on *APOE* genotype as homozygote *APOE3* allele (e3/e3) and *APOE4* carriers (e3/e4 and e4/e4).

### sPDGFRβ

Plasma sPDGFRβ levels were determined blinded to clinical and genetic data using an in-house developed MSD assay as we reported previously [43]. Briefly, each well of a 96-well 1-spot SECTOR® plate (Catalog No. L15XA-3, MSD) was coated with 0.2 µg of anti-human PDGFRβ goat polyclonal antibody (Catalog No. AF385, R&D Systems, Minneapolis, MN) in 5 µl 0.01 M phosphate-buffered saline (PBS) pH 7.4 + 0.03%Triton X-100 capture antibody. The plates were kept in a fume hood on a flat surface to air-dry the capture antibody solution overnight at room temperature. The sealed antibody-coated plates were kept at 4 ºC until use. To measure the levels of sPDGFRβ in plasma, the capture antibody-coated wells were first blocked with 150 µl of 1% Blocker B (Catalog No. R93BB-2, MSD) in 0.01 M PBS + 0.05% Tween-20 (PBS-Tween 20 wash buffer) for 60 min at 25 ºC with shaking on an orbital plate shaker at 500 rpm. The plate was washed with 200 µl/well PBS-Tween 20 three times and tapped on Kimwipes (Catalog No. 34133, Kimberly-Clark Worldwide, Inc.) to remove residual wash buffer prior to the addition of samples and standards. A carrier-free recombinant extracellular domain of human PDGFRβ protein (Catalog No. 385-PR/CF, R&D Systems) with concentrations ranging from 100 to 6400 pg/mL was used to generate a standard curve. Each human plasma sample was diluted 30-fold in 0.2% Blocker B in PBS-Tween 20 wash buffer, and 25 µL of the prepared standards and samples were pipetted into the designated wells. The plates were sealed and incubated overnight at 4 ºC with shaking on an orbital plate shaker at 500 rpm. The detecting antibody solution was prepared by combining anti-human biotinylated goat polyclonal antibody (Catalog No. BAF385, R&D Systems) and Sulfo-tag labeled streptavidin (Catalog No. R32AD, MSD), both at a concentration of 1 µg/ml, in 0.2% Blocker B containing PBS-Tween 20. The plate was washed with 200 µl/well PBS-Tween-20 three times, then tapped on Kimwipes to remove residual wash buffer before adding 25 µl of the detecting antibody solution. The plates were sealed after the addition of the detecting antibody and incubated at 25 ºC for 90 min with shaking on an orbital plate shaker at 500 rpm. The plate was washed with 200 µl/well PBS-Tween 20 three times, tapped on Kimwipes to remove residual wash buffer. MSD read buffer T (Catalog no. R92TC-3, 2X in ultrapure distilled water) was added in each well, and the plate was read immediately on MSD SECTOR Imager 6000 with electrochemiluminescence detection. The plasma sPDGFRβ assay was free from matrix effects, showing dilution linearity of the electrochemiluminescence signal when plasma samples were diluted 20 to 40-fold. The plasma assay for sPDGFRβ showed robust analytical performance consistent with the previously reported assay for CSF sPDGFRβ [43]. Using a common sPDGFRβ recombinant protein calibration standard, the assay has a lower limit of quantification (LLOQ) of 100 pg/mL, upper limit of quantification (ULOQ) of 26,000 pg/mL, and intra- and inter-assay coefficient of variation (CV) < 6%.

### Aβ peptides

Plasma Aβ_42_ and Aβ_40_ concentrations were determined using C2N Diagnostics’ immunoprecipitation liquid chromatography-tandem mass spectrometry-analytical method (PrecivityAD®) as reported previously [44].

### pTau217

A Meso Scale Discovery (MSD) S-PLEX human Tau (pTau217) assay (Catalog No. K151APFS) was used to determine plasma levels of phosphorylated Tau at threonine 217.

### Statistical analysis

All study variables were screened for outliers through visual inspection of variable distributions and removal of influential outliers greater than ± 3 standard deviations from the overall sample mean.

All participants had valid plasma sPDGFRβ values, but there were missing data for VRF for 1 participant, plasma Aβ_42/40_ ratio for 4 participants, and plasma ptau217 for 3 participants.

A priori planned comparisons of *APOE4* carriers vs. *APOE3* homozygotes with and without cognitive impairment on plasma sPDGFRβ levels were conducted using 2 × 2 (*APOE4* status x CDR score) ANCOVAs with Fisher least significant difference (LSD) pairwise comparisons with alpha < 0.05 after False Discovery Rate (FDR) correction for multiple comparison adjustment. Covariates were age, sex, education, and VRF burden. Where significant differences were observed, additional models correcting for age, sex, education, VRF burden and plasma AD biomarkers of ptau217 or Aβ_42/40_ were evaluated. Statistical analyses were performed in R and SPSSv29.

## Results

**Fig. 1:**
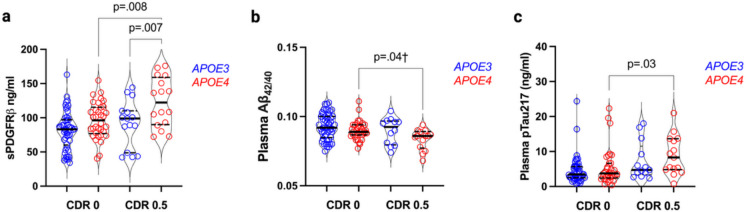
Plasma sPDGFRβ levels, plasma Aβ_42/40_ ratio, and plasma pTau217 levels in *APOE3* homozygotes and *APOE4* carriers cognitively unimpaired and with cognitive impairment. Plasma sPDGFRβ levels (**a**) plasma Aβ_42/40_ ratio (**b**) and plasma pTau217 levels (**c**) in cognitively unimpaired *APOE3* homozygotes (n = 50) and *APOE4* carriers (n = 34) carriers with Clinical Dementia Rating (CDR) score of 0, and in *APOE3* homozygotes (n = 15) and *APOE4* carriers (n = 16) carriers with cognitive impairment and CDR score of 0.5. Data are shown as individual points with mean value ± SD. All values are controlled for age, sex, education and VRF burden. All results are from 2 × 2 ANCOVAs with planned Fisher LSD pairwise comparisons with FDR correction as explained in the Methods. **†**Not significant after FDR-correction

Using an in-house developed MSD assay as described earlier (Sweeney et al. 2020), we show that plasma sPDGFRβ levels were significantly increased by 27% (p = 0.008) and 34% (p = 0.007) in *APOE4* carriers with cognitive impairment (CDR = 0.5) compared to *APOE4* carriers that are cognitively unimpaired (CDR = 0) and compared to cognitively impaired *APOE3* homozygotes (CDR = 0.5), respectively (Fig. 1a). On the other hand, there was no significant difference in plasma sPDGFRβ levels between cognitively impaired and unimpaired *APOE3* homozygotes. We also show that plasma Aβ_42/40_ ratio was significantly lower in cognitively impaired compared to unimpaired *APOE4* carriers (p = 0.04), although this difference does not survive FDR correction (Fig. 1b). Plasma Aβ_42/40_ ratio remains similar in cognitively impaired compared to unimpaired *APOE3* homozygotes (Fig. 1b). In contrast, plasma pTau217 levels were significantly higher in cognitively impaired compared to unimpaired *APOE4* carriers (p = 0.03), and again were not different between cognitively impaired compared to unimpaired *APOE3* homozygotes (Fig. 1c). In all these planned comparisons, all values are corrected for age, sex, education and VRF burden, and all p-values survived FDR correction.

**Fig. 2:**
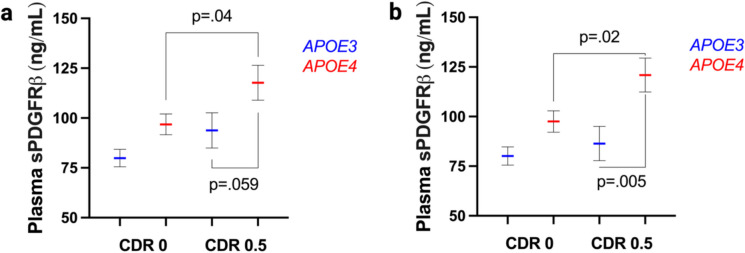
Plasma sPDGFRβ levels after controlling for plasma Aβ_42/40_ ratio and plasma pTau217 levels. (**a**) Estimated marginal mean ± standard error of measurement (SEM) of plasma sPDGFRβ after controlling for age, sex, education, VRF burden and plasma Aβ_42/40_ levels and (**b**) in a separate model after controlling for age, sex, education, VRF burden and plasma pTau217. All individual data are taken from Fig. 1 and the results are from 2 × 2 ANCOVAs with Fisher LSD pairwise comparisons as explained in the Methods

Next, we show the differences in sPDGFRβ levels between groups remain when controlled for plasma Aβ_42/40_ ratio (Fig. 2a), although differences between impaired (CDR 0.5) *APOE4* carriers versus *APOE3* homozygotes become a non-significant trend (p = 0.059). Findings also remained essentially unchanged after controlling for plasma pTau217 levels (Fig. 2b). Again, in all these comparisons all values were additionally corrected for age, sex, education and VRF burden.

## Discussion

The present study shows that plasma sPDGFRβ reflecting vascular BBB/pericyte cell damage is elevated in *APOE4* carriers with cognitive impairment (CDR = 0.5) compared to unimpaired *APOE4* carriers (CDR = 0), and relative to cognitively impaired *APOE3* homozygotes (CDR = 0.5) which remains significant after correcting for demographic variables (e.g., age, sex, education and VRFs). This finding is consistent with a previous report showing elevated CSF levels of sPDGFRβ in cognitively impaired *APOE4* carriers compared to unimpaired *APOE4* carriers and cognitively impaired *APOE3* homozygotes [20]. Interestingly, a previous CSF study was able to detect slightly increased levels of sPDGFRβ in cognitively unimpaired *APOE4* carriers compared to unimpaired *APOE3* homozygotes [20], which we were not able to reproduce here with plasma sPDGFRβ.

The present data show that plasma sPDGFRβ remains elevated in cognitively impaired *APOE4* carriers relative to cognitively unimpaired *APOE4* carriers and impaired *APOE3* homozygotes even after controlling for demographic variables and plasma Aβ_42/40_ ratio or plasma pTau217. Additionally, we show that plasma Aβ_42/40_ ratio was significantly lower in cognitively impaired compared to unimpaired *APOE4* carriers, although this difference did not survive FDR correction. Another study in cognitively healthy Super-Seniors showed that Aβ_42/40_ ratio was lower in *APOE4* carriers compared to non-*APOE4* carriers, but the difference did not remain significant after adjusting for demographic variables [45]. On the other hand, we showed that pTau217 levels remain significantly elevated in cognitively impaired compared to unimpaired *APOE4* carriers, and this difference survived FDR correction. In a study in cognitively healthy Super-Seniors, it has been also shown that after adjusting for demographic variables, plasma pTau181 was the only biomarker that remained significantly associated with *APOE4* status [45].

It has been reported that CSF sPDGFRβ levels are increased in MCI individuals [14, 20, 21, 32, 36] and in AD patients [30, 31, 37]. It has been also shown that sPDGFRβ correlated positively with increased BBB permeability determined by dynamic-contrast enhanced (DCE)-MRI in MCI individuals [14, 20, 21], and CSF albumin reflecting BBB breakdown [30–32, 36]. It has been also found that there is an age-dependent increase in CSF sPDGFRβ [31] as well as that CSF sPDGFRβ was positively associated with pTau181 and total tau [30, 31, 34–37] independently of CSF Aβ_42_ [31, 35] or amyloid brain deposits detected by positron-emission tomography [35]. It has been suggested that the natural trajectories of sPDGFRβ and AD biomarkers indicate that pericyte injury is an early event during transition from normal status to AD, even earlier than Aβ deposition [31]. The fact that sPDGFRβ levels correlate with cognitive dysfunction in *APOE4* carriers, independent of the early-stage AD biomarker ptau217, provides further support for disease models where neurovascular unit dysfunction is an early vascular mechanism of *APOE4*-associated AD risk that is distinct from later *APOE4* effects on amyloid and tau. Current recommendations for the diagnosis of AD include other markers beyond amyloid and tau, including markers of inflammation and neurodegeneration [46]. Further efforts should seek to establish biofluid sPDGFRβ levels, and possibly imaging markers of BBB permeability, as additional biomarkers important in the pathophysiology of AD. Longitudinal studies of plasma and CSF sPDGFRβ, and neuroimaging markers of BBB permeability, together with the current suite of amyloid, tau, and other biomarkers important in the pathophysiology of AD would aid in achieving this goal. These studies will also provide key data for establishing optimal cutoff values with high sensitivity and specificity for pericyte/BBB injury, facilitating clinical translation and diagnostic utility.

Another study found that CSF sPDGFRβ and markers of neuroinflammation are associated with AD biomarkers and differ by race, suggesting that vascular dysfunction and neuroinflammation may precede cognitive decline and disease pathology in the very early preclinical stages of AD, and there are race-related differences in these relationships [33]. It has been also found that changes in some components of the renin-angiotensin system correlated positively with CSF sPDGFRβ levels and CSF albumin in AD patients, suggesting that dysfunction in renin-angiotensin system is related to capillary dysfunction in AD [47].

The present study suggests that plasma sPDGFRβ is a useful biomarker of brain pericytes/BBB damage in *APOE4* carriers, and that elevated plasma sPDGFRβ levels are associated with cognitive dysfunction in *APOE4* carriers. These findings are consistent with prior findings reported for CSF sPDGFRβ [20]. The relative contribution of sPDGFRβ originating from the central nervous system (CNS) and peripheral sources in the systemic circulation, and the cut-off values for plasma sPDGFRβ, have yet to be determined. A significant correlation between serum and CSF sPDGFRβ levels has been reported previously [30]. Whether plasma sPDGFRβ can replace CSF sPDGFRβ as a marker of pericyte/BBB injury in other conditions including different stages of AD continuum and in relation to changes in established AD Aβ and tau biomarkers in plasma, CSF and brain remains to be determined by future studies.

## Data Availability

All raw data are deposited at USC RedCap data base and the corresponding authors will make them available upon reasonable requests.
